# Development of dual reporter vector system for estimating translational activity of regulatory elements

**DOI:** 10.1186/s12870-022-03735-1

**Published:** 2022-07-21

**Authors:** Aleksandra V. Suhorukova, Alexander A. Tyurin, Olga S. Pavlenko, Orkhan N. Mustafayev, Igor G. Sinelnikov, Irina V. Goldenkova-Pavlova

**Affiliations:** 1 Laboratory of functional genomics, Timiryazev Institute of Plant Physiology, Russian Academy of Sciences, Moscow, Russia; 2 Genetic resources institute, Azerbaijan National Academy of Sciences, Baku, Azerbaijan; 3 Laboratory of enzyme biotechnology, Federal Research Centre “Fundamentals of Biotechnology”, Russian Academy of Sciences, Moscow, Russia

**Keywords:** High throughput screening, Vector, Lichenase, *β*-glucuronidase, Regulatory elements, Transient expression

## Abstract

**Background:**

For the needs of modern biotechnology, a quantitative approach to the control of regulatory elements at all stages of gene expression has long become indispensable. Such a control regime is impossible without a quantitative analysis of the role of each regulatory element or pattern used. Therefore, it seems important to modify and develop the accuracy, reproducibility, and availability of methods for quantifying the contribution of each regulatory code to the implementation of genetic information.

**Results:**

A new vector system for transient expression in plants is described; this system is intended for quantitative analysis of the contribution of regulatory elements to transcription and translation efficiencies. The proposed vector comprises two expression cassettes carrying reporter genes (of the *Clostridium thermocellum* thermostable lichenase and *E. coli* *β*-glucuronidase) under the control of different promoters. Herewith we also propose a new method for quantification of the effect of tested regulatory elements on expression, which relies on assessment of the enzyme activities of reporter proteins taking into account the transcription of their genes.

**Conclusions:**

In our view, this approach makes it possible to precisely determine the amounts of reporter proteins and their transcripts at all stages of expression. The efficiency of the proposed system has been validated by the analysis of the roles of known translation enhancers at the stages of transcription and translation.

**Supplementary Information:**

The online version contains supplementary material available at (10.1186/s12870-022-03735-1).

## Introduction

The recent advance in the technologies for obtaining omic data has allowed for accumulation of tremendous array of information. Correspondingly, molecular biologists frequently need experimental verification of the biomolecular data. A wide range of reporter systems working in most different organisms have been designed for this purpose. For plants, the most relevant method is transient expression–agroinfiltration; it consists in the transfer of a large number of copies of vectors via the infiltration of the agrobacteria (*Agrobacterium tumefaciens*) carrying these vectors to the mesophyll of a model plant. The apparent advantages of this approach are rapidness, simplicity, and availability [1]. The vector systems optimized for the transient expression in plants commonly have the following specific features: the absence of a selective marker, the presence of silencing suppressor genes, and the fact that the reporter genes code for rapidly maturing proteins. This tool makes it possible to get precise data on the expression of target polypeptides in plants. Nonetheless, transient expression is the approach that depends on manifold parameters. A considerable set of factors, such as plant age and the conditions of plant growth, can add noise to the corresponding data [1].

In order to level the dependence of expression level on the uncontrolled conditions, we propose using the bireporter vector pLAUMe, carrying the genes of two reporter proteins, namely, *Clostridium thermocellum* thermostable lichenase (LicBM3) [2] and *E. coli* *β*-glucuronidase (GUS) [3, 4], which have shown a good performance as reporter proteins with different sensitivities being simple in use. This approach allows for a concurrent assessment of the expression levels of both the reporter genes under the control of a tested element and the gene being a kind of internal control. Thus, the presence of internal control module allows for assessment of the expression level of a gene from the target cassette despite the changes in external conditions. In addition, this makes it possible to avoid co-transformation, which considerably simplifies the experiment. The main advantages of the proposed vector system include simplification of the analysis of regulatory elements (no need in co-transformation) and a direct quantification of reporter proteins.

**Fig. 1:**
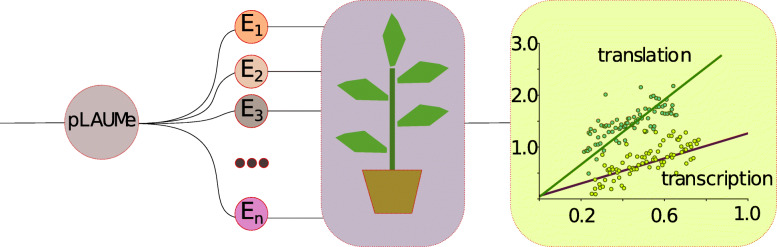
Scheme of the experiment. The experiment consists of three main stages: construction of plant expression vectors bearing tested enhancers; transient expression in plants and analysis of the expression levels of the reporter genes with linnear regression

In this work, we have designed and tested a vector system for assessing the efficiency of translation enhancers. Known and well-characterized enhancers were used for the analysis (discussed in Results section) [5–7]. In addition, we propose a new method for quantification of the effect of individual regulatory elements on expression based on assessment of enzyme activities of reporter proteins. We have demonstrated that it is feasible to measure a relative amount of reporter proteins and, as a consequence, to quantify the contribution of each enhancer to transcription and translation. The general plot of the experiment is shown in the Fig. 1.

## Results

### Model of vector system

**Fig. 2:**
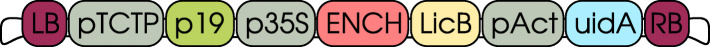
Scheme of the pLAUMe vector. p19 – Silencing supressor from tombusviruses. TCTP – arabidopsis translationally controlled tumor protein promoter. en35SCaMV –enhanced 35S CaMV promoter. LicB – lichenase gene. pAct – arabidopsis actin promoter. uidA – gene of beta-glucuronidase

**Fig. 3:**
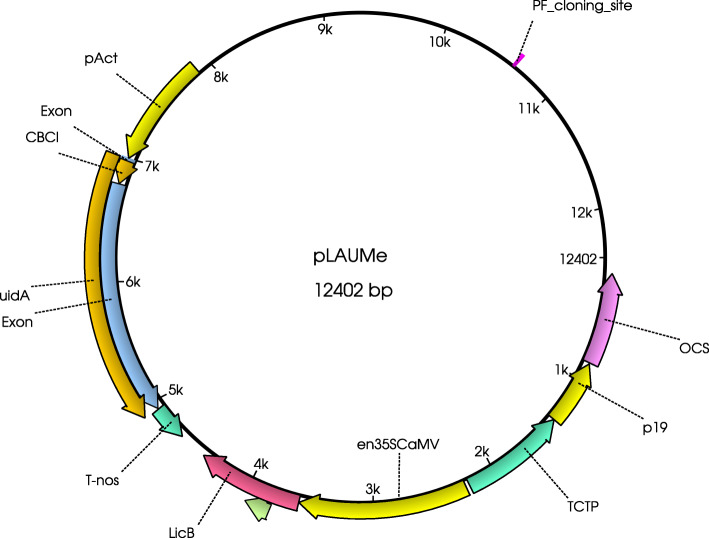
Map of the pLAUMe vector. OCS – octopin synthase terminator. p19 – Silencing supressor from tombusviruses. TCTP – arabidopsis translationally controlled tumor protein promoter. en35SCaMV –enhanced 35S CaMV promoter. LicB – lichenase gene. pAct – arabidopsis actin promoter. uidA – gene of *β*-glucuronidase. T-Nos – nopaline synthase terminator. CBCI – castor bean catalase intron. Data are based on [9]

Two reporter systems were united in the designed vector, namely, the *uid*A gene under the control of arabidopsis actin promoter, which acts as the internal control module, and the *lic*BM3 gene under the control of enhanced CaMV 35S RNA promoter, the test module for assessing the contribution of the studied regulatory elements (Fig. 2). In addition, the vector carries the cassette with the tombusvirus p19 silencing suppressor under the control of arabidopsis translationally controlled tumor protein promoter [7]. Being united in one vector molecule, these structural modules make it possible to avoid the co-transformation with individual vectors. The castor bean catalase intron and potato ST-LS1 intron were integrated into the *uid*A and *lic*BM3 genes, respectively, to prevent unauthorized expression in the intermediate prokaryotic systems. See Fig. 3 for the detailed map of the vector. The vector thus constructed was tested in *N. benthamiana* (the corresponding data are shown below with the results of testing of the remaining vectors). To verify the designed test system, we decided to use the already described and characterized enhancers (listed in Table 1). Note that for better coverage, we selected four short plant enhancers contrasting in their expression levels (deletion variants AT30, AT65, AT100, and AT208: AT5G46430, AT1G07260, AT1G67090, AT1G58420 of *A. thaliana*, respectively [7]), one long plant enhancer (GGR, geranyl-geranyl reductase enhanser) [5], and two synthetic enhancers (SynJ and SynM (MsynJ and SynM) [6]. The enhancers were integrated using CPEC (circular polymerase extension cloning) method [8], which allowed for a seamless integration of target fragments alone. Thus, the following expression vectors were obtained using the pLAUMe vector as the major component: pLAUMe-AT30, pLAUMe-AT65, pLAUMe-AT100, pLAUMe-AT208, pLAUMe-SynJ, pLAUMe-SynM, and pLAUMe-GGR, respectively. In these vectors, the sequence of a tested enhancer is located upstream of the *lic*BM3 reporter gene. To evaluate an impact of translational enhancer (TE) initial pLAUME (which does not contain any TE) vector was used as control group. Each of the constructed vectors was used to transform *A. tumefaciens* strain GV3101, which were further used for agroinfiltration *N. benthamiana* plants.

**Table 1:** Enhansers

enhancer	Description	Reference	Sequence	Length
AT30	Deletion varinat AT	doi:10.1093/nar/gkt864	CTCTAATCACCAGGAGTAAAA	21
AT65	Deletion varinat AT	doi:10.1093/nar/gkt864	GAGAGAAGAAAGAAGAAGACG	21
AT100	Deletion varinat AT	doi:10.1093/nar/gkt864	CACAAAGAGTAAAGAAGAACA	21
AT208	Deletion varinat AT	doi:10.1093/nar/gkt864	ATTATTACATCAAAACAAAAA	21
MsynJ	Modificated synthetic enhancer	http://www.biomedcentral.com/1472-6750/12/85	ACAGGCGCTATCAATCCGAAGCTAAACCATG	31
SynM	Modificated synthetic enhancer	http://www.biomedcentral.com/1472-6750/12/85	ACACGCTGGAATTCTAGTATACTTTTCCATG	31
GGR	Geranyl-geranyl reductse enhander	DOI 10.1007/s11248-013-9757-9	ACAAACTCAAAACACAGAGAACAGAGAGAG AGAGAGAGACTCCATTGTTGAAGTGTGTTC ACTGAACTCCTCCTCAAACAACA	83

### A comparative analysis of the functional activity of translation enhancers

**Fig. 4:**
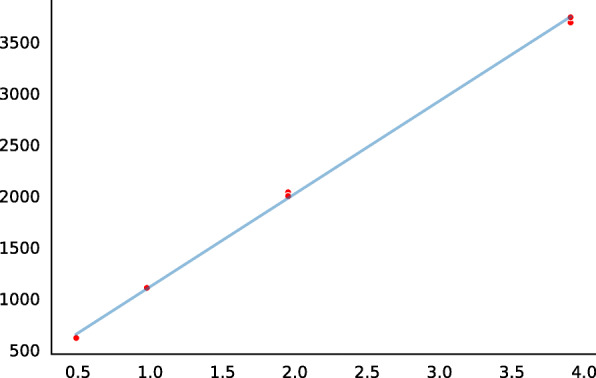
Standart curve for *β*-glucuronidase. Along the abscissa – concentration of 4-Methylumbelliferyl- *β*-D-glucuronide, nM; along the ordinata – fluorescence intensity

**Fig. 5:**
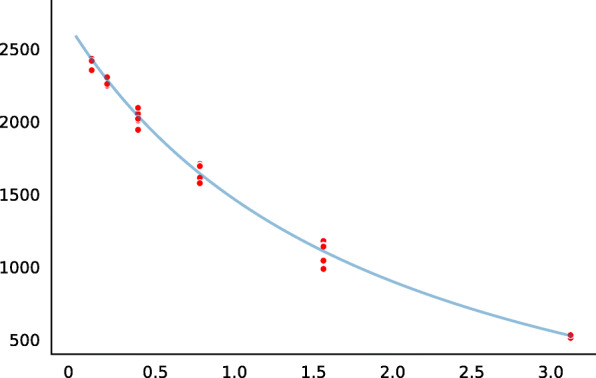
Standart curve for lichenase by enzyme. Along the abscissa – total amount of lichenase in the sample, ng; along the ordinata – fluorescence intensity

**Fig. 6:**
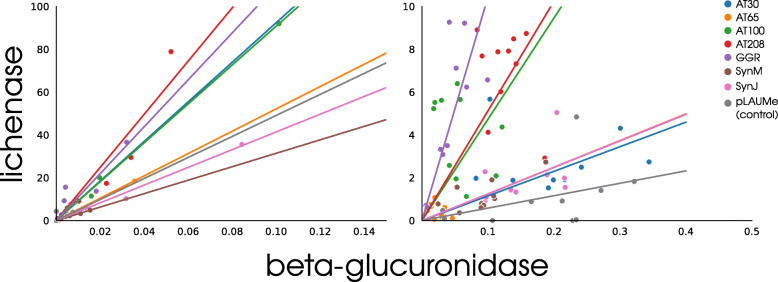
Left pane: comparison of reporter gene expression under the control of tested enhancers at the transcription stage (absolute expression is indicated for both lichenase and glucuronidase); Right pane : at the translation stage. For the reporter proteins, relative expression is shown. Lichenase activity is indicated in ng/ *μ*l of lichenase, *β*-glucuronidase activity – in nmol/ *μ*l of 4-Methylumbelliferyl- *β*-D-glucuronide

Total mRNA and total soluble protein were isolated in parallel from the agroinfiltrated leaf fragments to assess the expression of the reporter gene under the control of a tested regulatory element at the stages of transcription and translation. The transcription of reporter genes was assessed by qPCR in one experiment. The translational activities of reporter genes were analyzed according to the enzyme activities of reporter proteins (see Fig. 4 for beta-glucuronidase and Fig. 5 for lichenase). Using the above-described (Materials and methods) pipeline for data processing, we determined the regression slopes to construct the rating of the tested enhancers (Table 1). Analysis of the transcriptional (RT-qPCR; Fig. 6, left subplot) and translational (enzyme activities of reporter proteins; Fig. 6, right subplot) activities of the tested enhancers showed a linear dependence between the transcription and translation levels of the reporter genes. Table 2 lists the comparative data.

**Table 2:** ${LTR}_{e_{i}}$ - lichenase transcription (regression slope); ${LTL}_{e_{i}}$ - lichenase translation (regression slope); ${GTR}_{e_{i}}$ - glucuronidase transcription; ${GTL}_{e_{i}}$ - glucuronidase translation ; where *e*_*i*_∈{*AT*30,*AT*65,*AT*100,*AT*208,*GGR*,*SynM*,*SynJ*,*pLAUMe*}; the pLAUMe vector serves as control group

Enhancer	${LTR}_{e_{i}} $	*p*-value	${LTL}_{e_{i}}$	*p*-value	${LTR}_{e_{i}}/{LTL}_{e_{i}}$	$({LTR}_{e_{i}}/{LTL}_{e_{i}})/({LTR}_{pLAUMe}/{LTL}_{pLAUMe})$
AT30	923.9530	0.1699	11.4845	0.1177	0.0124	1.0524
AT65	521.4279	0.0009	12.4561	0.3780	0.0239	2.0226
AT100	907.7488	0.0001	47.2774	0.1903	0.0521	4.4097
AT208	1241.3535	0.0395	51.1762	0.1710	0.0412	3.4905
SynJ	413.8579	0.0037	12.4237	0.1199	0.0300	2.5417
SynM	314.1038	0.0047	12.3979	0.0178	0.0395	3.3419
GGR	1095.2883	0.0378	104.3354	0.0069	0.0953	8.0653
pLAUMe	490.8870	0.2697	5.7978	0.2651	0.0118	1.0000

## Discussion

Since the vector contains two reporter protein genes at once, the copy ratio (*lic*BM3/*uid*A) within T-DNA will always be 1:1. When expressed under controlled conditions (due to the fact that reporter genes are under the control of constitutive promoters), the ratio of expression levels (*lic*BM3/*uid*A) at the transcription stage, expressed in the number of mRNA copies, is also constant, and reflects some linear relationship (determined by the strength of the promoters). It is reasonable to assume that, other things being equal, the same ratio will be observed in relation to the target proteins (LicBM3/GUS). However, if one of the reporters is placed under the control of a regulatory element that influences the translation process, then this ratio (LicBM3/GUS) will change in the direction in which this regulatory element influences. Making a number of assumptions, we will consider this dependence to be linear. Then the ratio of the slopes of the straight lines (reflecting the ratios of mRNA reporters and their protein products) will demonstrate the contribution of this regulatory element to the control of translation.

We propose to evaluate the contribution of certain translational enhancers (both known and potential) according to the following scheme: i. Analysis of transcription of reporter genes using qPCR - determination of the absolute number of copies of the corresponding mRNA. ii. Analysis of the enzymatic activity of reporter proteins, using calibration curves built on a purified standardized enzyme - determination of the absolute number of copies of the corresponding enzymes. iii. Statistical analysis of the received data. Comparison of the slope of straight lines reflecting the ratio of mRNA (*lic*BM3/*uid*A) and proteins (LicBM3/GUS).

Thus, the presence of an internal control system physically associated with the main reporter gene and the tested regulatory element makes it possible to make the measurement of the reporter expression level under the control of this element more accurate, as well as significantly speed up and simplify the testing process itself due to the obsolescence of the cotransformation stage.

If we consider the currently existing vectors with several reporters in their composition, then bicistronic systems have recently become widespread. They are used to analyze the localization of a pathogen[10–12], study the activity of enzymes [13, 14], study heat shock proteins [15], transcription and post-transcriptional modifications [16, 17], study the activity promoters in bacteria [18], cell cycle [19], the important role of bicistronic systems in the analysis of IRES [20].

However, the bicistronic scheme is not quite suitable for the analysis of cis-regulatory elements.

Separately, it is worth returning to the stage of transcription analysis using qPCR, the need for this stage is dictated by the fact that, according to a number of studies [21, 22], some functional elements of the promoter can be located in the downstream direction relative to the transcription start point, which, in turn, leads to the inclusion these elements into the 5’-UTR. The opposite is also true, if the 5’-UTR in the distal region contains motifs similar to the functional elements of the promoter, then such 5’-UTRs can regulate not only translation but also transcription. Therefore, in our opinion, for the most accurate characterization of the tested translational enhancer, its role should also be analyzed at the transcription stage.

In this work, we propose to carry out a relative analysis of the role of translational enhancers, arbitrarily choosing one of them as a control. It should be noted that 5‘-untranslated regions often contain proximal functional elements (boxes) of the [21, 22] promoter, thus affecting the transcription process as well. Therefore, we propose to accurately assess the contribution of translational enhancers to the expression level by assessing changes in the ratio of reporter gene products both at the transcription and translation stages.

As mentioned above, we analyzed the expression levels of reporter genes under the control of the tested enhancers both at the transcription stage and at the protein accumulation stage. During qPCR data processing and reporter enzymatic activity analysis, we obtained 2 numerical vectors for each reporter gene:

- for lichenase transcription $LIC^{transcription}_{e_{i}}$;
- to translate lichenase $LIC^{translation}_{e_{i}}$;
- for glucuronidase transcription $GUS^{transcription}_{e_{i}}$;
- for translation of glucuronidase $GUS^{translation}_{e_{i}}$;

where *e*_*i*_∈{*AT*30,*AT*65,*AT*100,*AT*208,*GGR*,*SynM*,*SynJ*,*pLAUMe*} at the transcription stage and at the translation stage, the number of vector elements corresponds to the number of experimental variants (the number of tested enhancers plus control). Further, believing that some 5’-UTRs are able to determine the efficiency of not only translation, but also transcription, we normalized the expression vector at the translation stage for each reporter protein to a similar vector at the transcription stage, then the second normalization was carried out to the internal control vector (vector for gus), the order of normalization in this case is arbitrary due to the commutativity of multiplication. The resulting vector corresponds to some ratio of expression levels under the control of the studied translational enhancers. Thus, the final vector of the relative contribution of enhancers looks like this: $({LTR}_{e_{i}}/{LTL}_{e_{i}})/({LTR}_{pLAUMe}/{LTL}_{pLAUMe})$ (Table 2).

### Potential pitfalls

The presented vector system for evaluating the role of translational enhancers is not without drawbacks. As noted above, the use of a vector with physically linked reporter protein genes makes it possible to obtain values for the relative contribution of the studied cis enhancer to expression both at the transcription stage and at the translation stage. However, this is only true for samples collected at the same point in time. In view of the fact that reporter proteins may have, as we believe, different decay times, the ratio of their activities may change over time. We have not explored this point in sufficient detail. However, we assume that the construction of a time series for the ratios of activities amounts of reporter proteins can make it achievable to determine the true value of the contribution of the enhancer to the efficiency of translation. You should also be responsible in choosing a method for isolating proteins and RNA, because if the conditions are not met, it is quite easy to obtain biased estimates of the accumulation of reporter proteins.

## Conclusion

Within the framework of the presented study, a system for the relative assessment of the contribution of translational cis-regulatory elements was developed. For this, a vector was constructed for ranged expression in plants. Three main expression cassettes were included in this vector: p19 (silencing supresor), *lic*BM3 (reporter gene for evaluating the effect of the tested translational enhancer), *uid*A (reporter gene used for normalizing the expression of the target reporter (*lic*BM3)). In order to compare the effects of different cis-regulatory elements on translation, their contributions to both transcription and translation were evaluated. Comparison of the obtained results makes it possible to clearly identify the true impact of the translational enhancer, and the use of already known enhancers of different strength makes it possible to rank the studied regulatory elements.

## Materials and methods

### Plant growing

*Nicotiana benthamiana* plants were used in the work; the plants were grown on mineral wool using Knop’s solution according to the earlier described protocol [23]: photoperiod, 14 h of light/10 h of darkness and illumination, 130–150 *μ*Em^−2^s^−1^, temperature 22^∘^C and 60% humidity. The plants aged 6 weeks were used for agroinfiltration.

### Bacterial strains

The *A. tumefaciens strain* GV3101 transformed with individual constructs were grown for 48 h in the LB medium supplemented with 10 mg/L rifampicin, 30 mg/L gentamicin and 50 mg/L carbenicillin, and 100 mM of acetosyringone. In order to obtain a standardized lichenase preparation, *E. coli* strain XL1 Blue was transformed with the vector pQE30-LicBM3 (earlier developed by the team of the authors [24]).

### Construction of plant expression vectors

Standard molecular cloning procedures and PCR protocols were used. Restriction endonucleases, T4 DNA ligase, *Taq* and *Pfu* DNA polymerases, and phosphatases were used according to the manufacturers’ protocols (Promega, United States; Fermentas, Lithuania). The basic vector, named pLAUMe, was constructed in several steps. Initially, the *Sac*I/*Sma*I fragment carrying the reporter gene of thermostable lichenase, *lic*BM3, was cloned from the vector pQE-LicBM3 [24] to the vector pPGG 1A [25] hydrolyzed with *Sac*I and *Sma*I to get an intermediate vector, pPGG-L. The *Spe*I/*Xho*I fragment of pPGG-L, carrying the reporter gene *lic*BM3 under an enhanced CaMV 35S RNA promoter and terminator, was cloned into the vector pVIG-T [25] hydrolyzed with *Sac*I and *Sma*I to form the intermediate vector pGLR. At the next stage, the pACT-*uid*A-Tnos cassette, comprising *Arabidopsis thaliana* actin promoter, *E. coli* *β*-glucuronidase gene (*uid*A), and the *A. tumefaciens* termination sequence of the nopaline synthase gene, was synthesized (see Table S1 for the used primers). The pACT-*uid*A-Tnos cassette was cloned into the vector pGLR preliminary hydrolyzed with *Xho*I to get the vector pLAUMe. The last vector was further used to construct the vectors pLAUMe-SynM, pLAUMe-SynJ, pLAUMe-GGR, pLAUMe-AT30, pLAUMe-AT65, pLAUMe-AT100 and pLAUMe-AT208 by cloning the regulatory sequences between the CaMV 35S RNA promoter and *lic*BM3 reporter gene sequences using SLiC method [26]. A correct fusion of the genes with the corresponding regulatory sequences in the plant expression vectors pLAUMe-SynM, pLAUMe-GGR, pLAUMe-AT30, and pLAUMe-AT65 was confirmed by sequencing (Evrogen, Russia). See Supplementary Materials (Table S1) for detailed information.

### Agroinfiltration

Agroinfiltration followed the earlier described protocol [27]: *A. tumefaciens* cells of an overnight culture was centrifuged 20 min at 4000 ×g and suspended in the infiltration buffer (10 mM MES pH 5.5, 10 mM MgSO_4_, and 100 mM acetosyringone). For a typical assay, the leaves of greenhouse-grown *N. benthamiana* plants were infiltrated with the *Agrobacterium* mixture (approximately 5 mL/leaf) using a syringe without a needle. After the infiltration, the plants were further grown under greenhouse conditions. All experiments were performed in four to six replicates.

### Analyzing reporter gene transcription

The transcription of reporter genes was assessed using quantitative PCR in one experiment in five biological replicates and three technical replicates.

Total RNA from the majority of samples was extracted using TRIzol reagent (Evrogen, Russia) according to the manufacturer’s protocol. Prior to cDNA synthesis, RNA was treated with RNase-free DNase I (Thermo Scientific, United States) according to the manufacturer’s protocol to ensure no DNA contamination; then, the first-strand cDNA synthesis was carried out with approximately 2 *μ*g RNA using a Maxima H Minus First Strand cDNA Synthesis Kit (Thermo Scientific, United States) and oilgo-dT primers according to the manufacturer’s protocol. The primers were designed using PrimerBLAST (for *uid*A – F: CGGCAATAACATACGGCGTG; R: ATACCGAAAGGTTGGGCAGG; for *lic*BM3 – F: GGACCTTCGGACAACAATCCA; R: TCCTGGGAAGCATCGAATCC) with melting temperatures of 60^∘^C and amplicon lengths of 159 and 143 bp, respectively.

RT-qPCR was conducted in an Applied Biosystems QuantStudio 5 (Thermo Scientific, United States) using qPCRmix-HS SYBR (Evrogen, Russia). The reactions were performed in a total volume of 20 *μ*l of the reaction mixture containing 1 *μ*l of the template, 5 *μ*l of 5 × SYBR mix, 1 *μ*l of each specific primer to a final concentration of 200 nM under the following conditions: initial denaturation at of 95^∘^C for 180 s followed by two-step thermal cycling profile of denaturation at 95^∘^C for 15 s, and 40 cycles of combined primer annealing/extension at 60^∘^C for 30 s. No-template controls were included for each primer pair and each PCR reaction was completed in triplicate. To verify the specificity of the amplicon for each primer pair, a melting curve analysis was performed ranging from 60 to 95^∘^C with the temperature increasing steps of 1.6^∘^C /s at the end of each PCR run.

#### Constructing standard curves for copy number determination and absolute quantification

The pLAUMe (described above) vector carrying the *uid*A and *lic*BM3 genes was used as a standard. The standard sample was tenfold diluted to cover the concentration range of 0.2 to 200 ng/15 *μ*L. The absolute quantitative assay was performed using the Design Analysis Software v. 2.5.1 and Standard Curve v. 1.5.1 (Thermo Scientific, United States).

### Preparing lichenase standard

An overnight grown culture of *E. coli* strain XL1-Blue (Stratagene, United States) culture carrying the earlier produced vector pQE-LicBM3 [24] was diluted (1 : 50) with LB medium (Amresco, United States) and grown at 37^∘^C to an OD_600_ of 0.5. Then, the gene expression was induced with 1 mM isopropyl- *β*-D-1-thiogalactopyranoside (IPTG) to grow the culture at 28^∘^C for 48 h. The cells were separated from the medium by centrifugation for 15 min at 3160 ×g, washed twice with 50 mM Tris–HCl buffer pH 8.0, and suspended in the buffer containing 50 mM Tris–HCl pH 8.0, 10 mM EDTA, 0.1% Triton X-100, 5 mM DTT, 0.01% SDS, and 10 mM NaCl. The cells were incubated at 65^∘^C for 30 min and clarified by centrifugation for 30 min at 16 000 ×g and 4^∘^C. The supernatant was purified on a HisTrap HP column according to the manufacturer’s protocol (GE Healthcare, 17-5247-01). The eluted proteins were dialyzed against 5 mM Tris–HCl pH 8.0 at 4^∘^C and the purified thermostable lichenase protein was diluted in the buffer containing 20 mM Tris–HCl pH 7.4, 0.1 mM EDTA, 1 mM DTT, 200 *μ*g/mL BSA, 50% glycerol, and 100 mM KCl to a final concentration of 1 *μ*g/ *μ*L to use in the further experiments. The protein amount in preparations was determined using bicinchoninic acid (Sigma, United States) [28]. The proteins were separated by 12% SDS-PAGE according to Laemmli [29]. The molecular weight of proteins was determined using a Thermo Scientific PageRuler Unstained Protein Ladder (Thermo Fisher Scientific, Inc., United States).

### Preparing plant protein lysates

The leaves sampled from *N. benthamiana* plants on days 4–7 after agroinfiltration were pulverized in liquid nitrogen to a fine powder. Each powdered sample was suspended in three volumes of the 1 × PBS containing 0.5% Triton X-100 and incubated for 15 min at 4^∘^C and for 15 min at 50^∘^C. Cell debris was removed by centrifuging twice for 5 min at 16 000 ×g. The concentration of the samples was adjusted with 1 × reaction buffer. Translational activities of the reporter genes were measured in two independent experiments (eight to ten biological replicates each).

### Quantification of *β*-glucuronidase

*β*-Glucuronidase was quantified in plant extracts according to Jefferson et al. [3]. The amount of *β*-glucuronidase in preparations was determined using the calibration plot and expressed in nanomoles (4-MU) per unit volume per minute. To assume the amount of GUS protein in the plant samples we used curve constructed by standart *β*-glucuronidase diluted with factor 0.5.

### Quantification of licBM3 lichenase

For this purpose, lichenan at a concentration of 125 *μ*g/mL (if not stated otherwise) and Congo red solution at a final concentration of 0.005% were used. The fluorescence was assessed in a Synergy H1 (BioTek, United States) multimode microplate reader using 96-well microtiter plates [9].

### Statistical data processing and analysis

For this purpose, we wrote a special Python [30] script using several libraries, namely, pandas [31] for table data; NumPy [32] for data arrays; SciPy [33] and Scikit-learn [34] for statistical data processing; math for mathematical functions; and seaborn and matplotlib [35, 36] for data visualization. Data processing comprises the following stages: (i) Normalization of the samples according to dilution; (ii) Normalization of the samples according to volume; (iii) Construction of calibration curves; derivation of regression equation; (iv) Computation of the equation of dependence (inverse to regression equation); (v) Computation of absolute amounts of reporter proteins in unit volume; (vi) Derivation of linear regression equation for the dependence of amount of one reporter protein on the amount of another one for each tested enhancer; (vii) Regression analysis of quantitative PCR data; and (viii) Representation of the results of analysis as tables and plots. Construction of calibration curves for determining the amount of reporter proteins is described in the corresponding section of Materials and methods. The data were processed using linear regression. The equations for straight lines and calibration curves were optimized by least square technique with the help of Levenberg–Marquardt algorithm (using the Python SciPy library).

## Supplementary Information

**Additional file 1** Supplementary Tables.

## Data Availability

All datasets generated for this study are included in the paper/supplementary information.
